# Polyomaviruses KI and WU in Immunocompromised Patients with Respiratory Disease

**DOI:** 10.3201/1501.080758

**Published:** 2009-01

**Authors:** Thomas Mourez, Anne Bergeron, Patricia Ribaud, Catherine Scieux, Régis Peffault de Latour, Abdellatif Tazi, Gérard Socié, François Simon, Jérôme LeGoff

**Affiliations:** Hôpital Saint Louis, Paris, France

**Keywords:** polyomavirus, respiratory tract infection, stem cell transplantation, immune deficiency, dispatch

## Abstract

Polyomaviruses KI (KIPyV) and WU (WUPyV) were recently identified, mainly in respiratory specimens from children. Among 200 patients with respiratory disorders admitted to Saint Louis Hospital, Paris, France, KIPyV was detected in 8% and WUPyV in 1%. KIPyV was significantly more frequent among human stem cell transplant patients (17.8% vs. 5.1%; p = 0.01).

Recently, 2 new, distinct polyomaviruses (PyVs), KI (KIPyV) and WU (WUPyV), were identified in respiratory specimens, mainly from children <5 years of age with respiratory tract infections. The first retrospective studies of respiratory specimens in Sweden and Australia showed a KIPyV prevalence of 1% and 2.5%, respectively (*1*,*2*). Studies conducted in Australia and the United States showed a WUPyV prevalence in respiratory specimens of 3% and 0.7%, respectively (*3*). Further studies conducted in Canada and South Korea have shown similar frequencies (*4*,*5*). In this study, we examined the prevalence of KIPyV and WUPyV in immunocompromised patients with suspected respiratory tract infections.

## The Study

From January through June 2007, 265 respiratory samples were received in the laboratory of Saint Louis Hospital, Paris: 154 nasal aspirates (NA) and 111 bronchoalveolar lavage (BAL) specimens collected from 200 patients with suspected upper or lower respiratory tract infections. This hospital specializes in the management of immunocompromised patients. Respiratory samples were collected for the diagnosis of acute respiratory illness; 89% of samples were from immunocompromised patients. Their median age was 46 years (range 3.6–85.3 years). Given the observational nature of the study, French law did not require ethical approval or informed consent.

The specimens were routinely tested for influenza A and B viruses, respiratory syncytial virus, and parainfluenza viruses 1, 2, and 3 by immunofluorescence assay (Imagen; DakoCytomation, Trappes, France). Specimens positive for KIPyV or WUPyV were tested for adenoviruses; human bocavirus; human rhinoviruses; human metapneumovirus; human coronaviruses OC43, 229E, NL63, HKU1; and human PyVs BK and JC by using PCR methods (*6*–*11*). Total nucleic acid was extracted from 200 µL of NA, BAL, or stool specimens by using the EasyMag System (bioMérieux, Marcy l’Etoile, France). KIPyV was detected with an in-house real-time PCR assay targeting the *VP1* gene. The primers and hydrolysis probe were designed by using Primer Express 3.0 software (Applied Biosystems, Foster City, CA, USA). The final reaction volume was 25 µL and contained 12.5 pmol of SLKI-VP1s (5′-GGAAATACAGCTGCTCAGGAT-3′) and SLKI-VP1as (5′-CTTTGATACTTGAACCGCTTTCCTT-3′), 6.25 pmol of corresponding probe SLKI-VP1PR (5′-6FAM-CGTGACCCCACCCCTCATTACTGGTC-TAMRA-3′), 12.5 µL of TaqMan Universal Master Mix (Applied Biosystems), and 5 µL of DNA extract. The reaction was run on a 7500 Real-Time PCR System (Applied Biosystems). The specificity of positive specimens was confirmed by using PCR and nested PCR with primers POLVP1–39F/POLVP1–363R and POLVP1–118F/POLVP1–324R, as described (*1*). The PCR products were then sequenced and compared with the previously described sequences from Sweden and Australia (GenBank accession nos. EF127906, EF127907, EF127908, EF520287, EF520288, and EF520289). WUPyV was detected by PCR as described (*3*). PCR products with the expected molecular weights were sequenced by using primers AG0044 and AG0045 and compared to published sequences (GenBank accession nos. EF444550, EF444551, EF444552, EF444553, and EF444554) (*3*).

KIPyV was detected in 17 (6.5%) of the 265 respiratory samples and in 16 (8.0%) of the 200 patients. All cases were confirmed by a nested PCR targeting another region of the VP1 gene. Twelve of the 17 PCR products were successfully sequenced, and all shared 100% homology with published sequences. WUPyV was detected in only 2 patients (1.0%). Genome sequencing showed 98% homology with reported WUPyV sequences.

Six KIPyV-positive patients (37.5%) had co-infections with other respiratory viruses, and 2 of them (12.5%) had a pulmonary bacterial infection (Appendix Table). One WUPyV-infected patient who exhibited acute respiratory failure had concomitant pneumonia caused by *Pseudomonas aeruginosa* infection. None of the 15 patients who were positive for KIPyV or WUPyV and tested for fungi had respiratory or blood samples positive for *Aspergillus* spp.

The clinical characteristics of the patients with KIPyV or WUPyV infection and their general outcome until December 2007 are given in the Appendix Table. All but 3 had well-identified systemic immunosuppression. However, all had severe coexisting conditions. All KIPyV- and WUPyV-positive patients had acute respiratory disorders.

Eight KIPyV-positive patients had received allogeneic stem cell transplants; 5 of them had exhibited profound neutropenia in the 2 weeks before the respiratory sample was found to be positive. The detection of KIPyV was significantly more frequent among hematopoietic stem cell transplant (HSCT) recipients than among other patients (17.8% [8/45] vs. 5.1% [8/155], p = 0.01) (Table). Lung or sinus imaging was assessed by computed tomography scan for 12 KIPyV-positive patients. Lung parenchyma abnormalities were noted in 9 patients, and sinusitis was diagnosed for 2 patients.

**Table:** Frequency of KI polyomavirus detection in respiratory samples*

Sample source	KIV detection in respiratory samples, no. (%)		Total
	Positive	Negative	
HSCT patients	8 (17.8)†	37 (82.2)	45
Other patients	8 (5.2)	147 (94.8)	155
Total	16	184	200

*HSCT, hematopoietic stem cell transplantation. †χ^2^ test with Yates correction; p = 0.01.

Taking into consideration both the frequency of digestive symptoms in our patients and the former published detection of KIPyV in a stool sample, we looked for KIPyV infection in the available stool samples while the respiratory samples were being assessed for KIPyV (*1*). Strikingly, 3 of 4 samples tested were positive for KIPyV (with cycle threshold values of 35.0, 38.3, and 40.3). All were collected from HSCT recipients. In addition, 1 HSCT patient who experienced diarrhea demonstrated persistent excretion of KIPyV in 9 consecutive stool specimens collected between June and the end of November 2007 (data not shown).

## Conclusions

This study shows the prevalence of KIPyV and WUPyV among immunocompromised patients with respiratory disorders. Previously, these 2 viruses had been observed mainly in young children (*1*–*3*). Of the few adult patients with KIPyV or WUPyV infection mentioned in these studies, most were immunocompromised (*3*,*12*).

Considering the seemingly higher prevalence of KIPyV in our population (8%), immunocompromised patients may be more susceptible to this PyV, as they are to JC and BK viruses (*13*–*15*). Results from previous reports suggest a similar frequency of both KIPyV and WUPyV infections being found in respiratory specimens, ranging from 1% to 3%. In contrast, in our series, we found a likely difference between the prevalence of KIPyV (8%) and WUPyV (1%), which suggests that the replication or reactivation of the 2 viruses in the respiratory tract may differ between immunocompromised and immunocompetent patients. However, this difference requires further investigation, in particular, by using similar real-time PCR assays. Notably, a significantly higher prevalence of KIPyV infection was found among HSCT patients, which suggests that a profound T-cell deficiency may be a factor in facilitating KIPyV replication.

As reported in other populations, our patients who yielded positive specimens for KIPyV or WUPyV had conditions ranging from a common cold to acute respiratory distress that required invasive ventilation. Respiratory co-infections, observed in other studies, had likely accounted for at least some clinical features. In the 7 of our patients in whom KIPyV was the sole pathogen detected in the respiratory tract, despite comprehensive screening for viruses, bacteria, parasites, and fungi, clinical and radiographic patterns were varied. Some of the patients had only upper respiratory tract infections, notably sinusitis, whereas others had lung parenchyma abnormalities as defined by computed tomographic scan imaging. However, due to the retrospective nature of the study, and therefore the lack of a control group of immunocompromised patients without respiratory symptoms, the association of KIPyV infection with the occurrence of respiratory disease cannot be stated definitively.

In conclusion, the seemingly higher frequency of KIPyV shedding in immunocompromised patients (as observed with other PyVs) and the detection of KIPyV as a single pathogen in respiratory disease (e.g., as cytomegalovirus recurrence can lead to pneumonia in immunocompromised patients) together support a reactivation hypothesis. Nevertheless, a reinfection hypothesis cannot be excluded due to immunocompromised patients’ increased risk of acquiring viral infection from exogenous sources

Controlled prospective studies of KIPyV shedding before and during immunosuppression will help determine the pathogenic role of this virus. The clinical implication of KIPyV detection in stools and the mechanisms underlying the concomitant presence in gastrointestinal and respiratory tracts also deserve further analysis.

## Supplementary Material

Appendix Table Clinical characteristics and outcome of patients with KI or WU polyomavirus identified during the study*
