# Supplementary material for: Polyomaviruses KI and WU in Immunocompromised Patients with Respiratory Disease
Source: Emerg Infect Dis. 2009 Jan;15(1):107–9. doi: 10.3201/1501.080758 (PMC2662633; doi:10.3201/1501.080758)
Supplement: Appendix Table — Clinical characteristics and outcome of patients with KI or WU polyomavirus identified during the study* [file 08-0758_appT-s1.pdf]

Publisher: CDC; Journal: Emerging Infectious Diseases
Article Type: Dispatch; Volume: ; Issue: ; Year: ; Article ID: 08-0758
DOI: ; TOC Head:

Appendix Table. Clinical characteristics and outcome of patients with KI or WU polyomavirus identified during the study*

Sample no.	Age. y/sex	Date sample collected	Sample type	Ct value	Underlying disease	Type of transplant/date	Neutropenia in the past 2 weeks?†	Immunosuppressive treatment	Respiratory symptoms	Systemic symptoms	Digestive symptoms	Lung CT scan/sinus CT scan	Respiratory co-infections	Outcome of acute respiratory event	General outcome
KI polyomavirus															
SL2-39	29/F	2007 Jan 26	BAL	35.9	Unclassified immune deficiency	None	No	No	Cough, dyspnea	Fever, myalgia	None	Bibasal alveolar opacities/ND	*Haemophilus influenzae*	Cured	Alive 2007 Jun 21
SL2-47	27/F	2007 Jan 27	NPA	37.3	ALL	Unrelated bone marrow/2007 Jan 26	Yes	Methotrexate Cyclosporine	Epistaxis	Fever	None	ND/bilateral maxillary sinusitis	None	Cured	Alive 2007 Sep 05
SL2-78	14/M	2007 Feb 5	NPA	39.4	T-cell ALL	Genoidentical bone marrow/2007 Feb 15	Yes	Cyclosporine	Cough, hypoxemia	Fever	None‡	Diffuse centro-lobular micronodules/ND	hRV, HCoV-NL63 hRV, hMPV	Cured	Alive 2007 Dec 6
SL3-27		2007 Feb 19	NPA	38.8											
SL3-09	73/F	2007 Feb 13	NPA	37.0	Chronic cardiac failure	None	No	No	Cough, dyspnea, hypoxemia	Fever	Abdominal pain	ND (normal chest radiograph)/ND	None	Cured	Lost to follow-up
SL3-14	39/M	2007 Feb 14	NPA	35.4	Chronic EBV-related lymphoid proliferation	None	No	No	Cough, rhinorrhea, pharyngitis	None	None	Diffuse right ground glass opacities/ND	hRV	Cured	Alive 2007 Oct 11
SL3-83	36/M	2007 Mar 16	NPA	21.5	ALL, Grade 4 GVHD	Genoidentical bone marrow/ 2008 Feb 8	No	Prednisone (1.5 mg/kg/d), cyclosporine	Cough	Fever, shivers	Diarrhea, abdominal pain‡	Normal/bilateral maxillary sinusitis	None	NA	Died 2007 Apr 18§
SL3-84	72/M	2007 Mar 16	NPA	35.7	Chronic renal insufficiency, bronchiectasies	Kidney/2005 Oct	No	Mycophenolate mofetil, cyclosporine	Cough, sputum, rhinorrhea	Fever, myalgia	None	Thickened bronchiectasis/ND	hRV	Cured	Alive 2007 Jun 25
SL3-85	72/F	2007 Mar 16	NPA	37.0	Severe respiratory insufficiency	None	No	Prednisone (0.5 mg/kg/d)	Dyspnea, hypoxemia, rhinorrhea, pharyngitis	None	None	ND (normal chest radiograph)/ND	IFA	Cured	Alive 2007 Aug 10
SL3-97	59/M	2007 Mar 22	NPA	34.0	Severe respiratory insufficiency	None	No	No	Dyspnea, sputum, hypoxemia	None	None	ND (normal chest radiograph)/ND	HCoV-NL63	Cured	Alive 2007 Jul 17
SL3-99	26/M	2007 Mar 26	NPA	40.0	Aplastic anemia, dyskeratosis	Unrelated cord blood/2007 Feb 13	Yes	Prednisone (0.75 mg/kg/d)	None	Fever, shivers	None	Diffuse left nodules/ND	JCV, BKV, *Staphylococcus haemolyticus*	Progression¶	Died 2007 May 25#
SL4-11	19/F	2007 Apr 3	NPA	25.0	ALL	2 unrelated blood cords/2006 Sep 28	No	Prednisone (10 mg/d), cyclosporine	Cough, sputum	Fever	None	ND (normal chest radiograph)/ND	None	Cured	Alive 2007 Sep 11
SL4-14	5/F	2007 Apr 2	NPA	36.3	ALL	None	Yes	No	Rhinorrhea	Fever	Diarrhea	ND/ND	None	Cured	Alive 2007 Oct 10
SL4-17	27/M	2007 Apr 2	NPA	38.9	AML	Unrelated peripheral blood stem cell/2006 May 2	No	Prednisone (1 mg/kg/d)	Dyspnea	None	None	Diffuse alveolar opacities/ND*	None	Cured	??
SL4-39	28/F	2007 Apr 17	BAL	38.1	Systemic lupus erythematosus	None	No	No	Cough, sputum	None	None	Diffuse air trapping/ND	None o	Cured	Alive 2007 Jun 8
SL4-93	42/M	2007 Jun 5	NPA	29.9	AML, chronic extensive GVHD	Unrelated bone marrow/2007 Jan 18	No	Prednisone (0.25 mg/kg/d), cyclosporine	Sputum	Fever	None	Apical right alveolar opacity/ND	None	Cured	Alive 2007 Sep 19

Publisher: CDC; Journal: Emerging Infectious Diseases
Article Type: Dispatch; Volume: ; Issue: ; Year: ; Article ID: 08-0758
DOI: ; TOC Head:

SL4-94	17/M	2007 Jun 5	NPA	25.6	T-cell ALL	2 unrelated blood cords/ 2007 May 3	Yes	Prednisone (1 mg/kg/d)	Dyspnea	None	None §	Diffuse centro-lobular nodules/ND	PIV	Cured	Alive 2007 Nov 6
WU polyomavirus															
SL4-89	46/M	2007 Jun 2	NPA		T-cell lymphoma	None	No	No	Cough, dyspnea	None	None	Diffuse alveolar opacities and nodules/ND**	None	Progression of lung lymphoma	Alive 2007 Oct
SL4-100	44/F	2007 Jun 11	BAL		AML	None	No	No	NA (MV)	Fever	None	ND (bilateral opacities on chest radiograph)/ND	*Pseudomonas aeruginosa*	Cured	Alive 2007 Sep 2

*Ct, cycle threshold; CT, computed tomography; BAL, bronchoalveolar lavage; ND, not done; NPA, nasopharyngeal aspirate; ALL, acute lymphoblastic leukemia; hRV, human rhinovirus; HCoV, human coronavirus; hMPV, human metapneumovirus; EBV, Epstein-Barr virus; GVHD, graft versus host disease; IFA, influenza virus A; JCV, JC polyomavirus; BKV, BK polyomavirus; AML, acute myeloblastic leukemia; PIV, parainfluenza virus; NA, not applicable; MV, mechanical ventilation;
†WBC < 0.5×10^9/L.
‡Patients with KIV positive stools
§Death due to GVHD, aspergillosis, and multiple organ failure.
¶Invasive pulmonary aspergillosis diagnosed 2007 Apr 4; former nodules on CT scan had resolved while new micronodules appeared in URL.
#Due to aspergillosis and transplant rejection.
**No new abnormability compared with known lung disease.
